# Mitochondria-cytoskeleton associations in mammalian cytokinesis

**DOI:** 10.1186/s13008-016-0015-4

**Published:** 2016-03-18

**Authors:** E. J. Lawrence, E. Boucher, C. A. Mandato

**Affiliations:** Department of Anatomy and Cell Biology, McGill University, Montreal, QC Canada

**Keywords:** Mitochondria, Cytoskeleton, Cytokinesis, Microtubules, Actin, Miro, KIF5B

## Abstract

**Background:**

The role of the cytoskeleton in regulating mitochondrial distribution in dividing mammalian cells is poorly understood. We previously demonstrated that mitochondria are transported to the cleavage furrow during cytokinesis in a microtubule-dependent manner. However, the exact subset of spindle microtubules and molecular machinery involved remains unknown.

**Methods:**

We employed quantitative imaging techniques and structured illumination microscopy to analyse the spatial and temporal relationship of mitochondria with microtubules and actin of the contractile ring during cytokinesis in HeLa cells.

**Results:**

Superresolution microscopy revealed that mitochondria were associated with astral microtubules of the mitotic spindle in cytokinetic cells. Dominant-negative mutants of KIF5B, the heavy chain of kinesin-1 motor, and of Miro-1 disrupted mitochondrial transport to the furrow. Live imaging revealed that mitochondrial enrichment at the cell equator occurred simultaneously with the appearance of the contractile ring in cytokinesis. Inhibiting RhoA activity and contractile ring assembly with C3 transferase, caused mitochondrial mislocalisation during division.

**Conclusions:**

Taken together, the data suggest a model in which mitochondria are transported by a microtubule-mediated mechanism involving equatorial astral microtubules, Miro-1, and KIF5B to the nascent actomyosin contractile ring in cytokinesis.

**Electronic supplementary material:**

The online version of this article (doi:10.1186/s13008-016-0015-4) contains supplementary material, which is available to authorized users.

## Background

Mitochondria are essential organelles that function in ATP energy production, calcium buffering, and the generation of reactive oxygen species (ROS) [1–4]. The distribution of mitochondria within the cytoplasm is determined by interactions with the cytoskeleton. In mammalian cells, mitochondria are transported over long distances on microtubules, while short-range movement and docking require actin [5–7]. However, the role of the cytoskeleton and associated proteins in determining the distribution of mitochondria during cytokinesis, the final stage of cell division, is poorly understood.

Cytokinesis begins in anaphase with the assembly of a contractile ring composed of filamentous actin (F-actin) and Myosin II at the equatorial cortex. Constriction of the actomyosin ring results in a cleavage furrow that physically divides the parent cell into two daughter cells [8]. Microtubules of the mitotic spindle specify the assembly of the actomyosin ring by delivering the small GTPase RhoA to the equatorial cortex [9–11]. Subsequently, RhoA triggers local actin polymerisation and Myosin II contractility via downstream effectors such as the diaphanous-related formin mDia1, Rho kinase, and citron kinase [12–14].

We recently reported that mitochondria localize to the cleavage furrow during cytokinesis in mammalian cells using a mechanism that is dependent on microtubules of the mitotic spindle [15]. However, while we found that mitochondrial transport to the cleavage furrow during cytokinesis was microtubule-dependent, the subset of spindle microtubules and molecular machinery remained unknown. In contrast, a recent RNAi screen for new actin-based motors that function in division also identified the mitochondrial motor Myosin XIX (Myo19) as being involved in the regulation of mitochondrial distribution in dividing HeLa cells [16]. Indeed, when Myo19 expression was knocked down in dividing cells, mitochondria were mislocalized and asymmetrically inherited. The authors reported a similar phenotype for cells treated with Latrunculin B [16] and concluded that both Myo19 and actin were necessary for appropriate mitochondria distribution during cell division. In contrast, we previously showed, through systematic spatial and temporal analysis, that mitochondria accumulated at the cell equator following anaphase onset with no overall quantifiable differences in mitochondrial distribution between DMSO- or Latrunculin A-treated cells [15]. Thus, the mechanism of mitochondrial redistribution in dividing cells remains controversial.

The kinesin-1 family of motor proteins play a key role in transporting mitochondria in the anterograde direction along microtubules [17–20]. Mammals possess three isoforms of kinesin-1 (KIF5A, B and C). KIF5A and KIF5C are neuronal, whereas KIF5B is ubiquitously expressed [18]. KIF5B binds to mitochondria via an adaptor complex composed of the mitochondrial Rho GTPase (Miro) and the cytoplasmic adaptor protein Milton [5]. Miro is an highly conserved atypical GTPase that is anchored in the mitochondrial membrane [21–23]. Miro binds to Milton, which in turns binds to KIF5B, thereby linking mitochondria to microtubules [24, 25]. It is not known if the KIF5B-Miro machinery mediates microtubule-based transport of mitochondria to the furrow during cytokinesis.

Herein, we used a combination of live spinning disk and superresolution structured illumination microscopy (SIM) to gain mechanistic insights into the previously reported relationships of mitochondria with microtubules and actin in mammalian cytokinesis. The mechanism of microtubule-based transport of mitochondria to the furrow was investigated using dominant-negative mutants of KIF5B and Miro-1. Finally, systematic spatial and temporal quantification of control- and cell-permeable C3 transferase treated-cells, allowed us to investigate the relationship of mitochondria with the contractile ring during cytokinesis.

## Results

### Mitochondria associate with astral microtubules during cytokinesis

**Fig. 1:**
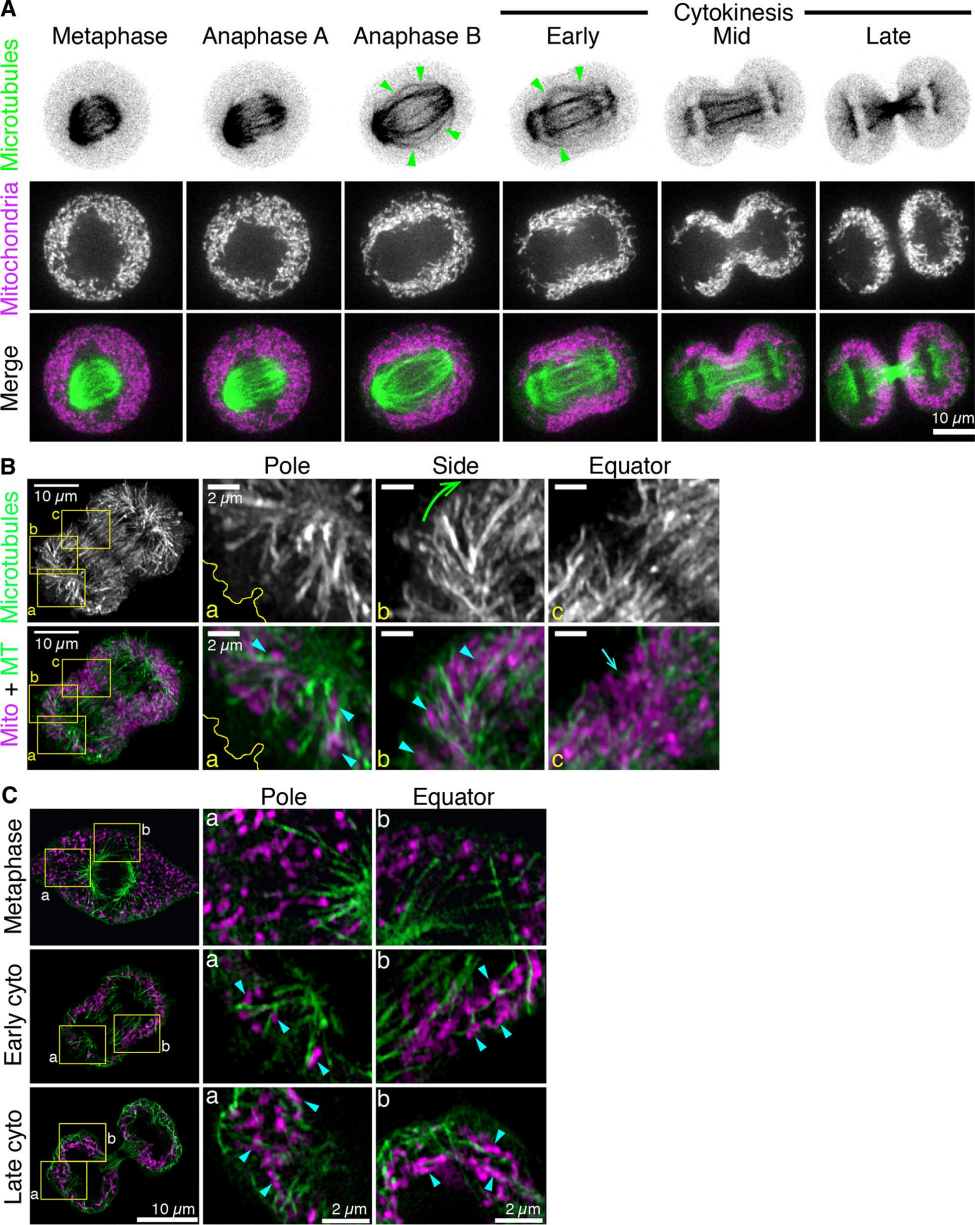
Imaging of microtubules and mitochondria in dividing HeLa cells. **A** Spinning disk confocal images of HeLa cells transfected with GFP-Tubulin and stained with MitoTracker Deep Red FM to visualize microtubules (shown in *green*) and mitochondria (shown in *magenta*) respectively. Images are maximum projections of five z-slices from the centre of the confocal stack at six representative time-points of division from metaphase to late cytokinesis. *Green arrowheads* indicate equatorial astral microtubules. (**B**, **C)** HeLa cells were stained for mitochondria (MitoTracker Deep Red FM) then fixed and stained for microtubules (anti-α tubulin, shown in *green*) and visualized by SIM. In (**B**) images are maximum projections of the full confocal stack. The *yellow* insets indicate the regions at the cell pole (**a**), side (**b**) and equator (**c**) that have been magnified in* adjacent panels*. *Blue arrowheads* indicate mitochondria associated with astral microtubules. The *green arrow* indicates the direction of equatorial astral microtubules curving towards the cleavage furrow and the *blue arrow* indicates mitochondria accumulated in a microtubule-devoid region at the equator. The *yellow* outline indicates the position of the cell cortex. In (**C**) images are central z slices from the confocal stack of cells in metaphase (*top row*), early cytokinesis (*middle row*) and late cytokinesis (*bottom row*). The* yellow insets* indicate regions at the cell pole (**a**) and equator (**b**) that have been magnified in* adjacent panels*. *Blue arrowheads* indicate mitochondria that are associated with microtubules in early and late cytokinesis

We previously demonstrated that mitochondria are transported to the cleavage furrow in a microtubule-dependent manner [15, 26]. To gain further insight into the relationship between mitochondria and microtubules during cell division and determine the specific subset of spindle microtubules responsible for transporting mitochondria to the furrow, we imaged microtubules and mitochondria in dividing HeLa cells by spinning disk confocal microscopy and superresolution SIM (Fig. 1). HeLa cells were transfected with GFP-Tubulin and stained with MitoTracker Deep Red FM to detect microtubules and mitochondria respectively. Images of microtubules (green) and mitochondria (magenta) at six representative stages of division from metaphase to late cytokinesis are given in Fig. 1A (for a full time series see Additional file 1: Movie S1). Mitochondria begin to accumulate at the cleavage furrow coincident with the appearance of equatorial astral microtubules of the mitotic spindle (Fig. 1A, green arrowheads). However, the visualization of astral microtubules was limited by the resolution of the spinning disk microscope. To overcome this, we imaged microtubules and mitochondria at high resolution using SIM. Mitotic HeLa cells were stained with an anti-alpha tubulin antibody and MitoTracker Red CMX Ros to visualize microtubules and mitochondria respectively (Fig. 1B, C). A maximum projection image of the full confocal stack of a representative cell in early cytokinesis and magnified regions at the pole, side and equator of the cell are given in Fig. 1B. Mitochondria at the cell poles were localized in proximity to the polar astral microtubules (Fig. 1B; inset a, blue arrowheads). In addition, long equatorial astral microtubules originating from the cell poles curved towards the cleavage furrow (Fig. 1B; inset b, green arrow) and appeared to be associated with mitochondria (Fig. 1B; inset b, blue arrowheads). Interestingly, mitochondria at the cleavage furrow were clustered in a region devoid of microtubules, likely occupied by the contractile ring (Fig. 1B; inset c, blue arrow). To better visualize the association of mitochondria with microtubules, we analysed single z slice images taken from the centre of the stack of cells in metaphase, early- and late-cytokinesis (Fig. 1C). In metaphase cells, mitochondria did not appear to be associated with microtubules at either the equator or the poles of the cell (Fig. 1C; top row). We also confirmed using Nocodazole that the metaphase mitochondrial distribution is not dependent on microtubules (Additional file 2: Figure S1). In contrast, mitochondria in early cytokinesis appeared to be associated with astral microtubules at both the equator and poles (Fig. 1C; middle row, blue arrowheads). In late cytokinesis, mitochondria also appeared to be associated with astral microtubules at the equator and the poles (Fig. 1C; bottom row, blue arrowheads). These observations suggest that astral microtubules are the subset of spindle microtubules responsible for transporting mitochondria to the cleavage furrow during cytokinesis.

### Miro-1 localizes with mitochondria at the cleavage furrow during cytokinesis

**Fig. 2:**
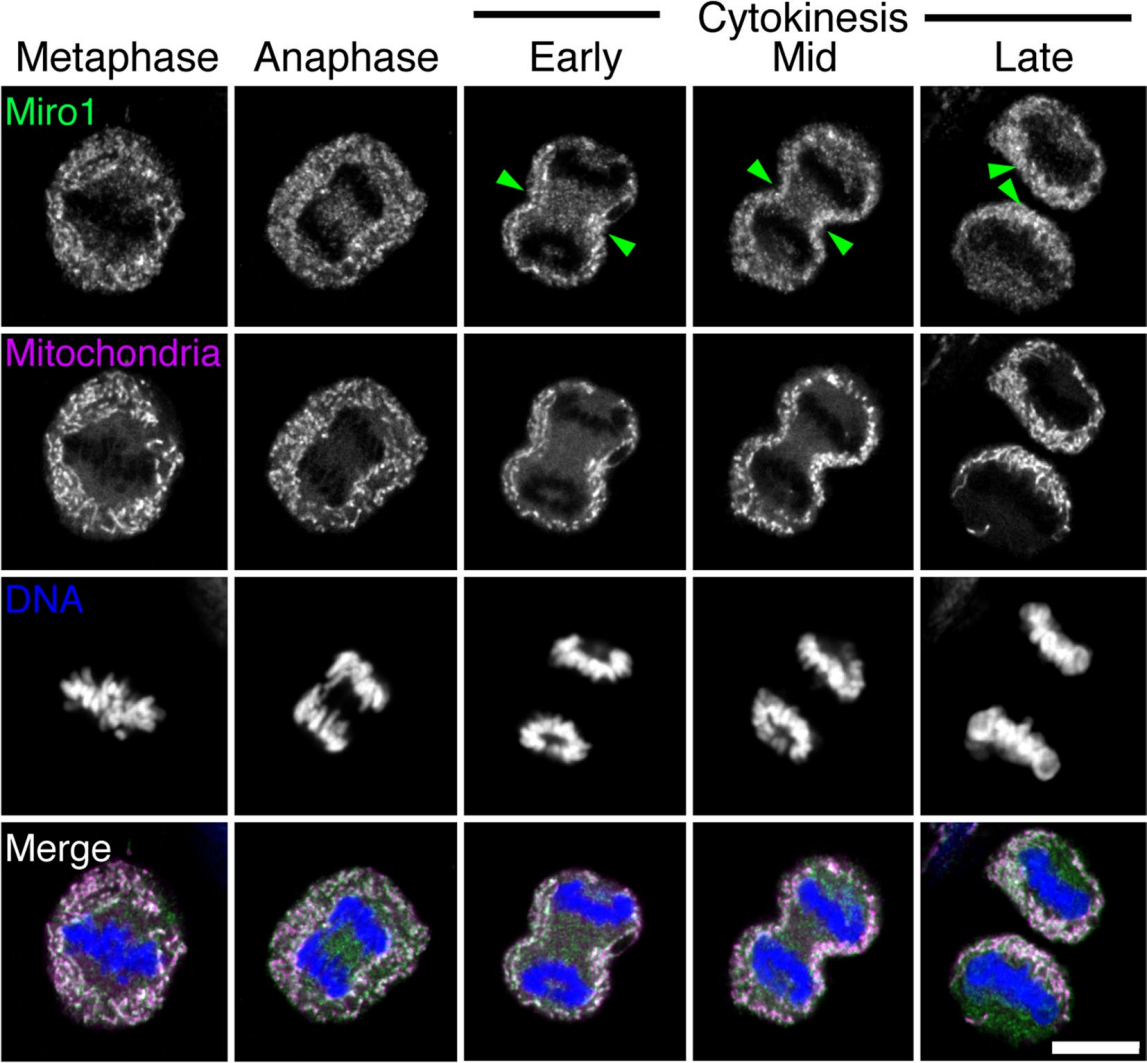
Visualization of Miro-1 distribution in dividing HeLa cells. Fixed confocal images of HeLa cells stained with MitoTracker Red, an anti-RhoT1 antibody and DAPI to visualize mitochondria (*magenta*), Miro-1 (*green*) and DNA (*blue*) respectively. Images are single z slices from the centre of the confocal stack of cells at five representative stages of division from metaphase to late cytokinesis. *Green arrowheads* indicate Miro-1 localized to the cleavage furrow. *Bar*, 10 μm

In line with our observation that mitochondria are transported along equatorial astral microtubules, we investigated the role of the Miro-Milton-KIF5B machinery in localizing mitochondria during cytokinesis. To visualize the distribution and localisation of Miro-1 during cytokinesis, HeLa cells were stained with MitoTracker Red, then fixed and stained with an anti-RhoT1(A16) antibody (shown in magenta) to detect Miro-1 (shown in green). Five representative stages of division from metaphase to late cytokinesis are shown in Fig. 2. In metaphase, Miro-1 was homogenously distributed in the cell cytoplasm. Cells in early-, mid-, and late-cytokinesis showed increased Miro-1 signal at the cleavage furrow (Fig. 2, green arrowheads). As expected, in all stages of division, the Miro-1 distribution colocalized with mitochondria (Fig. 2, merge), which is compatible with recently published observations by Kanfer et al. [27]. We also observed some signal that did not co-localize with mitochondria, this may represent some non-specific staining of the antibody, or a yet undescribed localisation of Miro-1.

### Miro-1 and KIF5B are required for mitochondrial distribution during cytokinesis

**Fig. 3:**
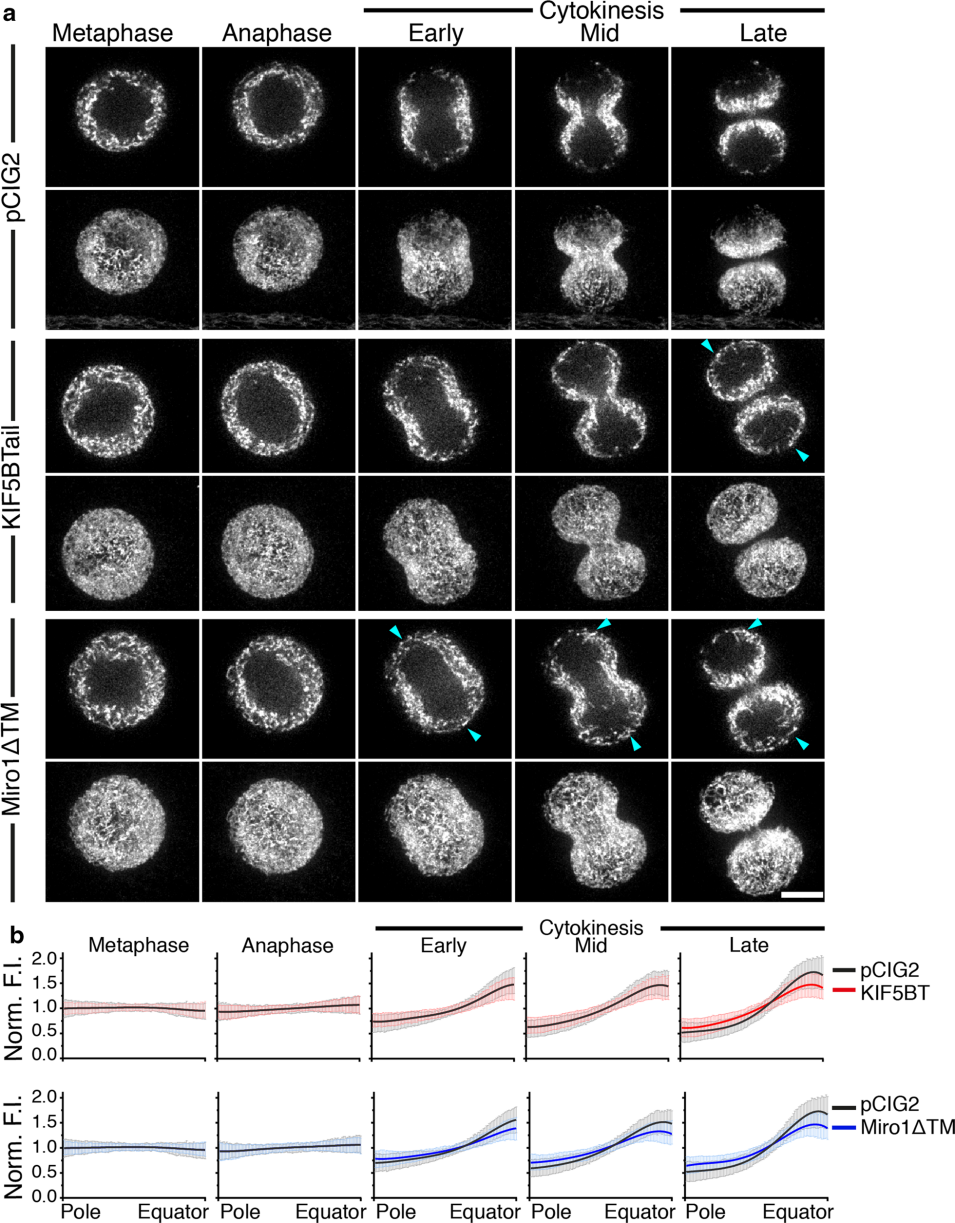
Visualization and quantification of mitochondrial distribution in dividing HeLa cells expressing KIF5BTail and Miro-1ΔTM. **a** Spinning disk confocal images of HeLa cells transfected with control vector (*top two rows*), KIF5BTail (*middle two rows*) or Miro-1ΔTM (*bottom two rows*) and stained with MitoTracker Deep Red FM to visualize mitochondria. Images are single z slices from the centre of the confocal stack and maximum z-stack projections are shown for five representative time points from metaphase to late-cytokinesis. *Blue arrowheads* indicate mitochondria that are mislocalized are the cell poles in KIF5BTail- and Miro-1ΔTM-expressing cells. *Bar*, 10 μm. **b** Quantification of mitochondrial fluorescence intensity from cell pole to equator in control (14 cells, N = 56), KIF5BTail- (14 cells, N = 56), and Miro-1ΔTM-expressing cells (16 cells, N = 64) during metaphase, anaphase, and early-, mid- and late-cytokinesis. The normalized distance from cell pole to equator is displayed on the *x-axis* and the average fluorescence intensity normalized against the mean is displayed on the *y-axis*. Data are represented as the mean ± SEM and lines fitted by non-linear regression. The difference in mitochondrial distribution was statistically significant in late cytokinesis in KIF5BTail-expressing cells (F-Test, p < 0.05) and from early cytokinesis onwards in Miro-1ΔTM-expressing cells (F-Test, p < 0.05) compared with control cells

To examine the role of Miro-1 and its binding partner, KIF5B, in cytokinesis, mitochondrial distribution was assessed in dividing HeLa cells transfected with either control vector, dominant negative Miro-1 (Miro-1ΔTM), or dominant negative KIF5B (KIF5BTail). Miro-1ΔTM lacks the transmembrane domain that anchors Miro-1 to the outer mitochondrial membrane [22] and, thus, exerts a dominant negative effect by binding and sequestering TRAK1 and KIF5B in the cytoplasm. KIF5BTail consists of the C-terminal cargo-binding domain of the full-length KIF5B and is able to bind mitochondria but not microtubules [28] and, thus, inhibits microtubule-based mitochondrial transport. Transfected cells were stained with MitoTracker Deep Red FM and imaged by spinning disk confocal microscopy. Images of five representative time points from metaphase to late cytokinesis in control, Miro-1ΔTM and KIF5BTail-expressing cells are given in Fig. 3 (for the full time series see Additional file 3: Movie S2). In control cells, mitochondria were enriched at the cleavage furrow and depleted from the cell poles during cytokinesis (Fig. 3a). In KIF5BTail-expressing cells, mitochondria were partially mislocalized at the cell poles in late cytokinesis (Fig. 3a). In Miro-1ΔTM-expressing cells, mitochondria were mislocalized at the cell poles from early-cytokinesis through to late cytokinesis (Fig. 3a). Furthermore, mitochondrial enrichment at the cleavage furrow was reduced in cells expressing Miro-1ΔTM. We quantified these observations by performing line-scan measurements of mitochondrial fluorescence intensity from cell pole to equator in control, KIF5BTail-, and Miro-1ΔTM-expressing cells at each representative phase of division (Fig. 3b). Quantification confirmed that the difference in mitochondrial distribution was statistically significant in late cytokinesis in KIF5BTail-expressing cells and from early cytokinesis onwards in Miro-1ΔTM-expressing cells compared with control cells. We also quantified the effect of wild-type, full-length Miro1 expression and observed no difference in mitochondrial distribution compared with control cells (Additional file 4: Figure S2). These results support a role for Miro-1 and KIF5B in mediating the interaction of mitochondria with microtubules during cytokinesis.

### Mitochondrial enrichment at the cell equator occurs simultaneously with the formation of the contractile ring

**Fig. 4:**
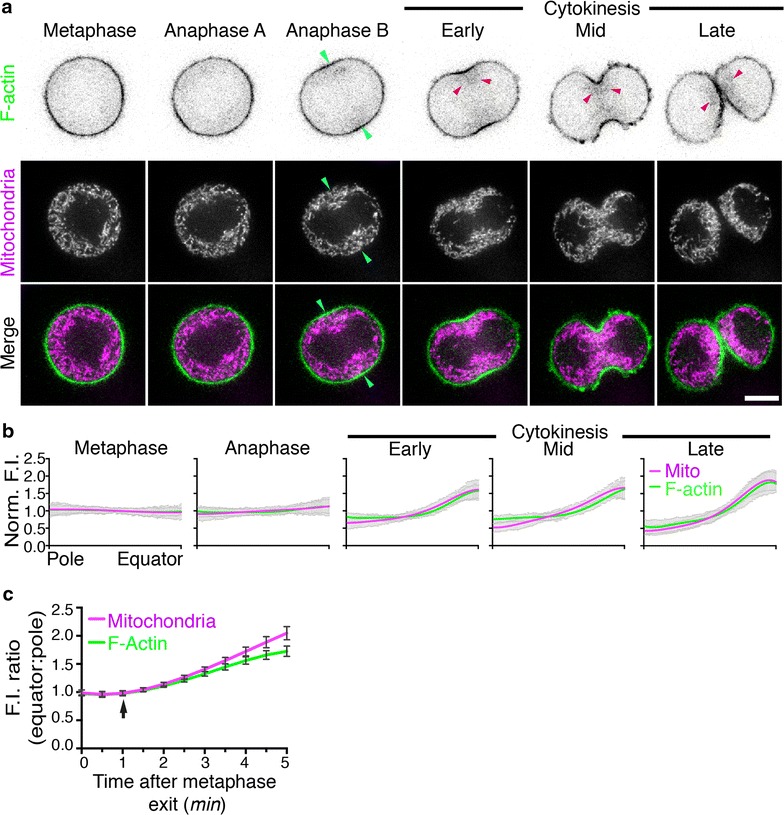
Visualization and quantification of F-actin and mitochondrial distributions in dividing HeLa cells. HeLa cells were transfected with GFP-UtrCH and stained with MitoTracker Deep Red FM to visualize F-actin (shown in *green*) and mitochondria (shown in *magenta*) respectively then imaged by spinning disk confocal microscopy. **a** Representative time-lapse images of a dividing HeLa cell showing F-actin and mitochondrial enrichment at the cell equator and depletion at the cell poles as division proceeds. Images are maximum projections of five z-slices from the centre of the confocal stack. *Green* and *magenta arrowheads* indicate F-actin and mitochondria localized to the cell equator respectively. *Blue arrowheads* indicate mitochondria colocalized with F-actin at the cell equator. *Red arrowheads* indicate enriched F-actin in the subcortical regions of cytokinetic cells. *Bar*, 10 μm. **b** Quantification of mitochondrial and F-actin fluorescence intensity from cell pole to cell equator at five representative stages of division. The normalized distance from cell pole to equator is displayed on the *x-axis* and the average fluorescence intensity normalized against the mean is displayed on the *y-axis*. Data were represented as mean ± SEM (8 cells, N = 32) and lines were fitted by non-linear regression. The polarization of mitochondria and F-actin towards the cell equator was statistically significant relative to metaphase from anaphase onwards (F-Test, p < 0.05). **c** Average equator: pole fluorescence intensity ratios (*y-axis*) were plotted against time following metaphase exit (*x-axis*) for both mitochondria (*magenta line*) and F-actin (*green line*) from metaphase exit (t = 0 min) to late anaphase (t = 5 min). Data were represented as mean ± SEM (8 cells, 4 quadrants; N = 32). The *black arrow* indicates the onset of mitochondrial and F-actin polarization towards the cell equator

In a previously published study, we used systematic spatial and temporal quantification of mitochondria distribution to show that mitochondrial transport to the equatorial portions of the cell equator in dividing cells was not affected by Latrunculin A or Jasplakinolide treatment [15]. However, these observations do not preclude that actin may act indirectly to regulate mitochondria distribution, for example through sequestration or for localized (i.e. at the furrow) transport. Indeed, a knockdown of the actin-binding based motor protein, Myo19, was shown to cause mitochondrial mislocalization to the cell poles distribution during cytokinesis [16]. An attractive explanation for this apparent discrepancy would involve a mechanism similar to that of neural cells where actin provides a docking mechanism following delivery the cell equator by microtubule-mediated transport [29–34]. For these reasons and to investigate further the relationship between mitochondria and actin, dividing cells were transfected with GFP-UtrCH, a marker for F-actin, stained with MitoTracker Deep Red FM to visualize mitochondria and imaged using spinning disk microscopy (Fig. 4). The distribution of F-actin and mitochondria at six representative stages of division from metaphase to late cytokinesis is shown in Fig. 4a (for the full time series see Additional file 5: Movie S3). Mitochondria enrichment at the cell equator coincided with the appearance of the contractile ring in anaphase B as indicated by cortical F-actin staining (Fig. 4a). Furthermore, mitochondria and cortical F-actin were progressively depleted from the cells poles as division proceeded. Interestingly, mitochondria appeared to co-localize with a cloud of sub-cortical F-actin that persisted throughout cytokinesis (Fig. 4a, red arrowheads). Spatial and temporal quantification of the pole: equator fluorescence intensity (F.I.) for both F-actin and mitochondria revealed that the polarization of mitochondria and F-actin occurred simultaneously as division proceeded (Fig. 4b). Indeed, no significant statistical difference was found between the pole: equator F.I. of mitochondria and F-actin in all stages of division. This colocalization of mitochondria with sub-cortical actin was also observed in cells displaying aberrant (i.e. collapsed or aggregated) sub-cortical actin morphologies, as those cells also displayed corresponding aberrant mitochondrial distribution (Additional file 6: Figure S3).

Next, we sought to quantify the time of onset of F-actin and mitochondria enrichment at the cell equator. To measure equatorial enrichment, equator: pole F.I. ratios for both F-actin and mitochondria were calculated at 30 s intervals following metaphase exit. The mean equator: pole F.I. ratio of eight cells (four quarters for each cells) ± SEM was plotted against time (Fig. 4c). Analysis revealed that the equatorial enrichment of both actin and mitochondria initiated at 1-min post-metaphase exit (Fig. 4c, arrow). Thus, the onset of mitochondrial enrichment at the cell equator occurs simultaneously with the onset of the formation of the actomyosin contractile ring.

### Inhibiting contractile ring formation prevents mitochondrial enrichment at the cell equator

**Fig. 5:**
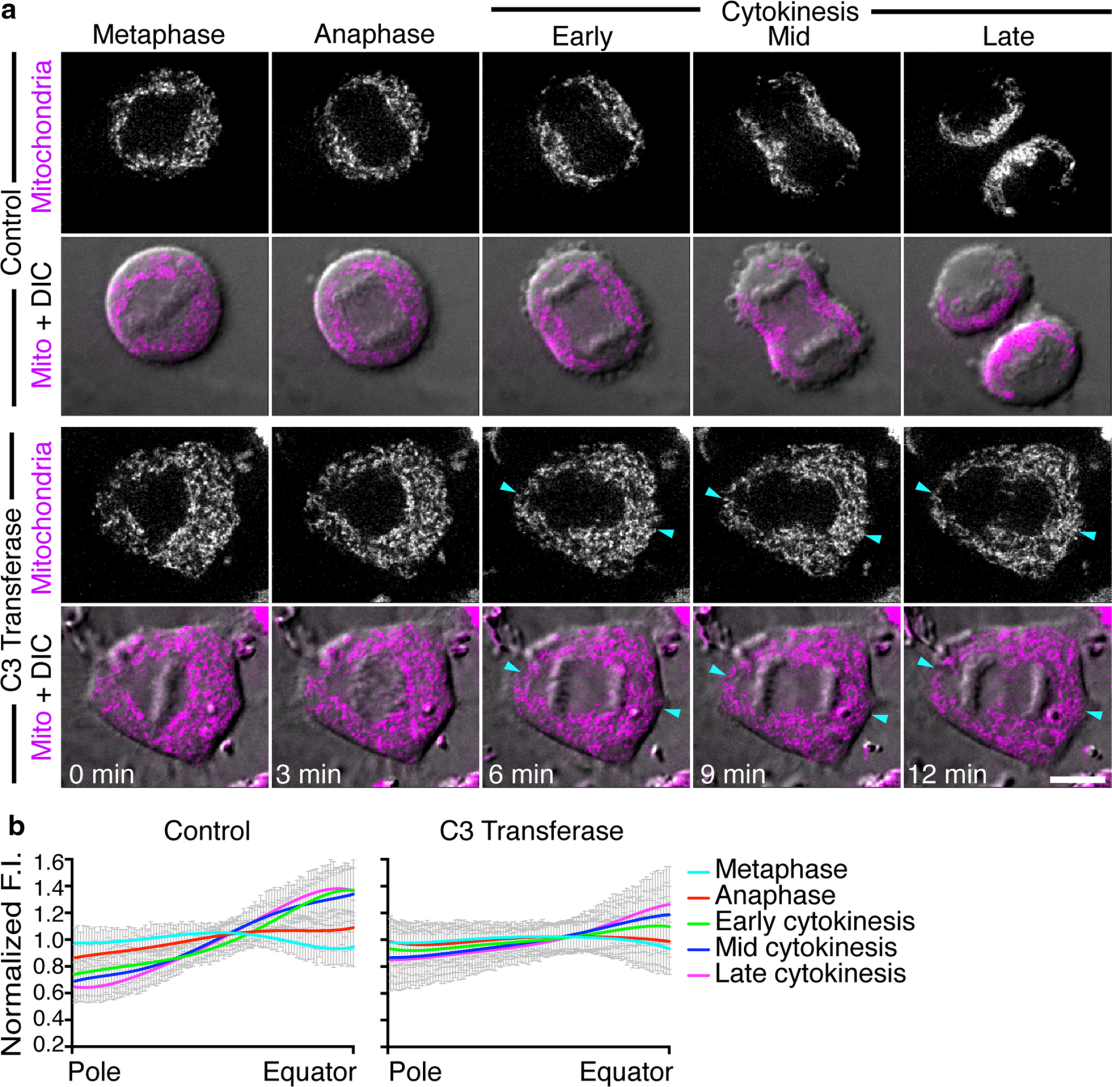
Visualization and quantification of mitochondrial distribution in control and C3 transferase-treated HeLa cells. **a** Spinning disk confocal images of HeLa cells treated with C3 transferase to inhibit RhoA activity and stained with MitoTracker Deep Red FM to visualize mitochondria (*magenta*). A single central z-slice and an overlay of the central slice with the corresponding DIC image are shown for five representative stages of division from metaphase to late-cytokinesis. *Blue arrowheads* indicate mitochondria mislocalized at the cell poles in C3-treated cells. *Bar*, 10 μm. **b** Quantification of mitochondrial fluorescence intensity from cell pole to cell equator in control (9 cells, N = 36) versus C3-treated (17 cells, N = 68) cells from metaphase to late cytokinesis. The normalized distance from cell pole to equator is displayed on the *x-axis* and the average fluorescence intensity normalized against the mean is displayed on the *y-axis*. Data are represented as the mean ± SEM and lines fitted by non-linear regression. The difference in mitochondrial distribution was statistically significant in C3-treated cells relative to control cells from early cytokinesis onwards (F-Test, p < 0.05)

The formation of the actomyosin contractile ring at the cleavage furrow is a spatiotemporally regulated event orchestrated by RhoA activity [9–14]. To investigate whether mitochondria enrich at the cell equator in the absence of a contractile ring, contractile ring formation was blocked by incubating cells with a commercially available, cell permeable, Rho-specific inhibitor, C3 transferase. C3 transferase is an ADP ribosyl transferase that selectively ribosylates RhoA, RhoB and RhoC proteins on asparagine residue 41, rendering them inactive. It has extremely low affinity for other members of the Rho family such as Cdc42 and Rac1 and does therefore not affect these GTPases. C3 transferase-treated cells were then stained with MitoTracker Deep Red FM and imaged by spinning disk confocal microscopy (Fig. 5). Images of five representative time points of division in control and C3-treated cells are given in Fig. 5a (for the full time series see Additional file 7: Movie S4). As expected, control dividing cells showed a progressive depletion of mitochondria from the cell poles to the equator and cleavage furrow. In contrast, RhoA inhibition caused cytokinesis failure and a reduction in mitochondrial polarization towards the cell equator (Fig. 5a, blue arrowheads). We confirmed these observations using the previously described systematic quantification method (Fig. 5b). Quantification confirmed that the difference in mitochondrial distribution was statistically significant in C3-treated cells from early cytokinesis onwards compared with control cells.

## Discussion

**Fig. 6:**
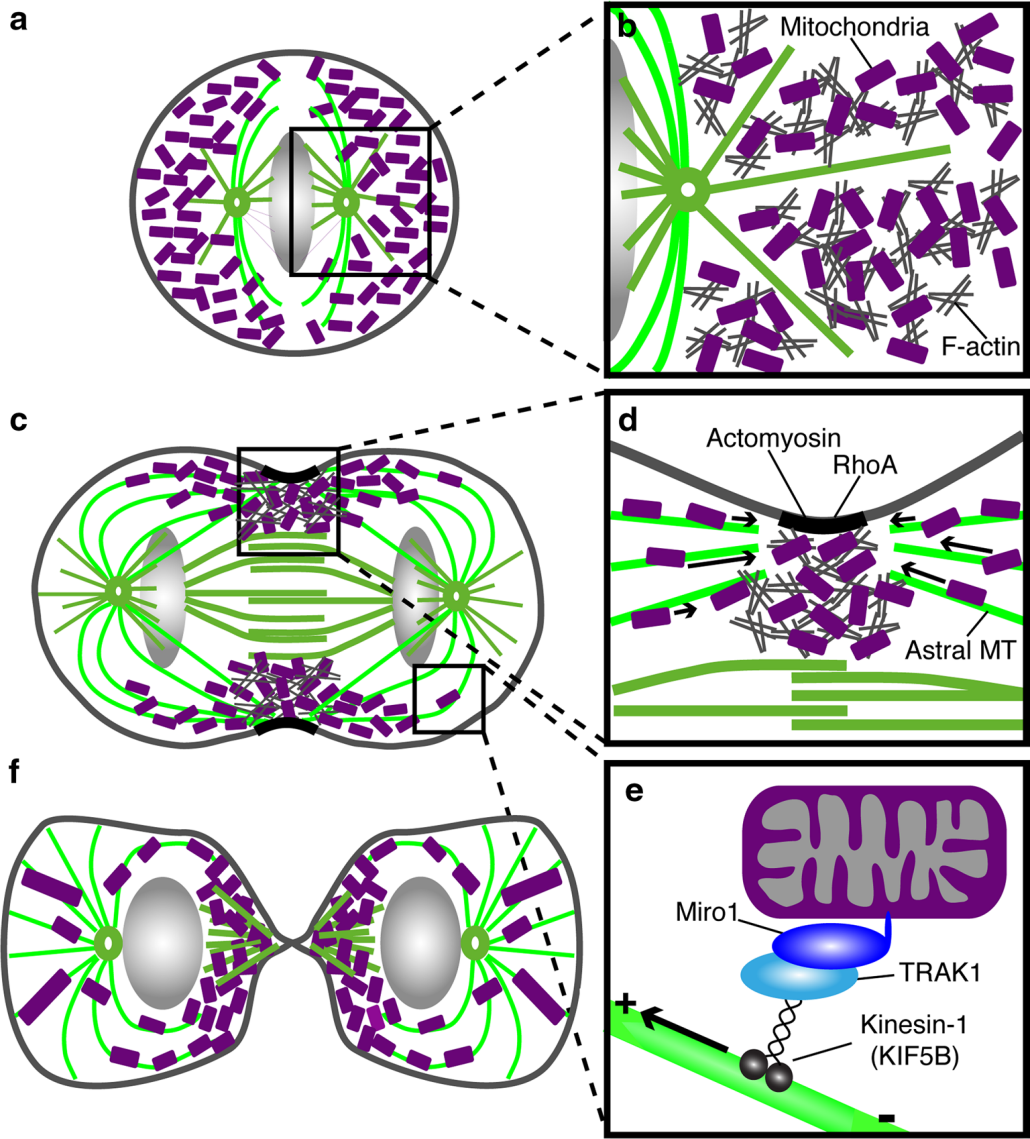
Model depicting the actin and microtubule-based regulation of mitochondrial distribution during cell division. **a** Mitochondria in metaphase are evenly dispersed in the cytoplasm. **b** The association of mitochondria with cytoplasmic F-actin may promote mitochondrial dispersal in metaphase. **c** During cytokinesis, mitochondria enrich at the cleavage furrow and are depleted from the cell poles. **d** Equatorial astral microtubules deliver mitochondria to the cleavage furrow. Following delivery on microtubules, mitochondria may dock to actin in the cleavage furrow. RhoA activity promotes mitochondrial localization to the furrow, possibly by triggering formin-mediated actin polymerization. **e** Miro-1 is required for transporting mitochondria to the plus ends of microtubules at the cleavage furrow, possibly via interaction with KIF5B. **f** In late cytokinesis, mitochondria begin to re-fuse and are transported away from the furrow towards the cell poles along microtubules in preparation for interphase

We previously reported that mitochondria localize to the furrow during cell division and that microtubule depolymerization inhibits this process [15]. In this study, we further validate these observations by demonstrating using superresolution microscopy that mitochondria appear to associate with astral microtubules of the mitotic spindle, are enriched at the cell equator coincident with the nascent contractile ring and require Miro-1 for proper distribution during cytokinesis (Fig. 6). A similar a model of microtubule- and Miro-1-dependent delivery of mitochondria to a contractile ring has been observed at the immune synapse in T-cells [35].

The involvement of microtubules and Miro-1 in the re-distribution of mitochondria during the transition from late cytokinesis to interphase has also been recently confirmed by Kanfer et al., which provided evidence that, upon entry into mitosis, CENP-F redistributes to mitochondria via Miro-1 binding and links mitochondria to microtubule plus-tips [27]. Herein, we investigated the role of KIF5B in linking mitochondria to microtubules during mitosis and found that inhibition of KIF5B activity caused mislocalized mitochondria in late cytokinesis but not during earlier stages of cytokinesis. One interpretation is that KIF5B mediates mitochondrial transport only during late cytokinesis. Alternatively, since our transfections were stringently optimized to minimize cytotoxic effects, dominant negative expression levels may not have been adequate to completely inhibit endogenous KIF5B activity. Another possibility is that other members of the kinesin-3 family members, such as KIF1B and KLP6, or CENP-F may compensate for KIF5B inhibition [36, 37]. In this regard, it would be interesting to assess the role of CENP-F and other kinesins in mediating the microtubule-mitochondria interaction at earlier stages of cytokinesis.

Following inhibition of Miro-1 activity and mitochondrial mislocalization, we did not observe obvious defects in cytokinesis or mitochondrial inheritance. This may be because mitochondria were only partially mislocalized and hence were still present, although reduced, at the furrow. It is also worth noting that our protocol did not allow for the observation of the effect of disrupted Miro-1/KIF5B over multiple generations, which may have yielded more robust mitochondrial mislocalization.

The fact that mitochondria polarize towards the cell equator simultaneously with contractile ring formation further supports, albeit indirectly, a microtubule-based mechanism of mitochondrial distribution during cytokinesis since microtubules are also required to stimulate contractile ring assembly at the equatorial cortex [38]. In addition, our data show that mitochondrial distribution is coordinated with a key event of cytokinesis, contractile ring assembly. Whether the enrichment of mitochondria in the vicinity of the contractile ring or retention of mitochondria at the furrow during ingression has functional consequences for cytokinesis remains to be established. While we previously reported that mitochondria are transported to the cleavage furrow via an actin-independent mechanism [15, 26], a recent study showed that depletion of the actin-based motor Myo19 by RNAi in dividing HeLa cells caused mitochondria to aberrantly localize to the spindle poles during cytokinesis and resulted in defects in mitochondrial inheritance and cytokinesis [16]. However, whether Myo19 anchors mitochondria to the contractile ring remains unknown since, in contradiction with our present and previous observations [15], they did not report mitochondria enrichment at the cleavage furrow in their control cells. The same group also failed to show enrichment of mitochondria to the equatorial portion of dividing cell in presence of Latrunculin B. The reason for this is unknown, but may relate to methodological differences in quantification methods, seeding density, or cell health. Indeed, we have reason to believe that the aberrant mitochondrial and F-actin distribution we sometimes observed during cell division in transfected cells (Additional file 6: Figure S3), may have been may be linked to altered cell shape or adhesion. Indeed, Fink et al. [39] suggested that adhesion geometry can bias dynamic subcortical actin structures in mitotic cells. Spindle positioning have been shown to be actin-dependent in a number of other models [40], and subcortical actin clouds and retraction fibres have been suggested to be involved in centrosome positioning [41]. Under our proposed model, aberrant spindle positioning due to aberrant retraction fibres (as seen in the aggregated phenotype of Additional file 6: Figure S3) would produce equally aberrant mitochondria distributions.

Interestingly, we also observed the colocalization of mitochondria with a subcortical pool of F-actin at the cleavage furrow. The source of this subcortical F-actin pool is unknown, but may represent pre-formed F-actin filaments that have been delivered to the cleavage furrow for incorporation into the contractile ring [38]. Alternatively, actin may be directly polymerized on the surface of furrow-enriched mitochondria. A recent paper by Li et al., showed the formation of actin filament on the outer mitochondrial membrane in interphase and mitotic cells [42]. Therefore, it is possible that actin at the cleavage furrow may promote mitochondrial docking and further enrich mitochondria at the furrow.

Mitochondria were retained at the cell poles and the enrichment of mitochondria at the cell equator was reduced when RhoA was inhibited. Interestingly, RhoA has previously been shown to reduce mitochondrial motility by promoting mitochondrial tethering to actin via mDia1-activation and local actin polymerization [43, 44]. In the context of dividing cells, local RhoA-triggered actin polymerization at the equatorial cortex in dividing cells may act similarly to inhibit mitochondrial motility in the vicinity of the cleavage furrow. However, it is difficult to discern the effects of RhoA inactivation since such cells exhibit a dramatically altered cell morphology. Additionally, since mDia1 has been shown to regulate microtubules at the furrow [45], we cannot exclude that mitochondrial mislocalization following RhoA inhibition may be due to the perturbation of microtubules at the furrow. This would however, still be compatible with microtubule-mediated transport of mitochondria to the cleavage furrow.

Finally, while there is now considerable evidence for microtubule-mediated transport to the cleavage furrow, it is also possible for mitochondria to be passively distributed. An example of such a mechanism would involve the mitochondria being squeezed toward the equator by the growth of the spindle in anaphase, telophase and early cytokinesis. Considering the importance of mitochondrial inheritance in ensuring daughter cell viability, it may be envisaged that mitochondria distribution during cell division relies on both passive and active, microtubule-mediated processes.

## Conclusions

Herein, we demonstrate, for the first time, an association of mitochondria with astral microtubules of the mitotic spindle and their co-localization with the nascent contractile ring in early cytokinesis. Furthermore, we provided evidence that mitochondrial localization to the furrow requires KIF5B and Miro-1 and the formation of a contractile array. In this model (Fig. 6), mitochondria are delivered to the cleavage furrow along equatorial astral microtubules in a mechanism involving the KIF5B/Miro-1 trafficking. A similar a model of microtubule-dependent delivery and actin-dependent docking at a contractile ring has been observed in the context of the immune synapse in T-cells [35].

Enriching mitochondria at the cell equator may contribute to the robustness of cytokinesis by promoting actomyosin assembly and contractility at the equatorial cortex. Cytokinesis failure leads to centrosome amplification and tetraploidy and is a hallmark of certain cancers [46, 47]; therefore, a complete understanding of the mechanisms underlying cytokinesis may have important therapeutic implications.

## Methods

### Cell culture and drug treatments

HeLa cells purchased from ATCC were maintained at 37 °C in a 5 % CO_2_ incubator and grown in Minimum Essential Media (MEM; Invitrogen) supplemented with 2 mM GlutaMAX (Gibco) and 10 % foetal bovine serum (FBS; Invitrogen). To stain for mitochondria, live cells were incubated for 15 min with 20 nM MitoTracker Red CMX Ros or MitoTracker Deep Red FM (Molecular Probes) as indicated in the text. To inhibit RhoA, cultured HeLa cells were incubated with 2 μg/ml of a commercially available, cell-permeable version of C3 transferase (Cytoskeleton Inc.) for at least 4 h prior to live cell imaging. C3 transferase is an ADP ribosyl transferase that selectively ribosylates RhoA, RhoB and RhoC proteins on asparagine residue 41, rendering them inactive [48]. It has extremely low affinity for other members of the Rho family such as Cdc42 and Rac1 and does therefore not affect these GTPases. Analysis was limited to metaphase cells exhibiting the hallmarks of RhoA inhibition: a spindly, non-round morphology [49, 50].

### Plasmid constructs

The cDNA encoding the wild-type, full-length human Miro-1 as well as Miro-1 lacking the transmembrane binding domain (Miro-1Δ593-618, hereafter referred to as Miro-1ΔTM) were purchased from Addgene (Addgene plasmids 47888 and 47895; originally published in [22]. The cDNA encoding the motor domain-less KIF5B (hereafter referred to as KIF5BTail) was a kind gift from C. Hoogenraad, Utrecht University, The Netherlands, and was successfully used to study microtubule trafficking [51, 52]. Miro-1ΔTM and KIF5BTail cDNA sequences were amplified by polymerase chain reaction using the Expand High Fidelity polymerase (Roche). Miro1ΔTM was amplified with the primer pair: 5′-CCGCTCGAGCGGATGAAGAAAGACGTGCGGAT-3′ and 5′-CCGGAATT.

CCGGTCAAAACGTGGAGCTCTTGAGGT-3′. KIF5BTail was amplified with the primer pair: 5′-CCGCTCGAGCGGATGTGGCGTAATGGGGAGACG-3′ and 5′-CCGGAATTCCGGTCAAGTTGGAGAAGCTGCTGGAT-3′. The underlined sequences correspond to Xho1 (forward primer) and EcoR1 (reverse primer) restriction sites. Full length Miro1, Miro1ΔTM, and KIF5BT were subcloned into a pCIG2-ires-eGFP bicistronic vector (a kind gift from M. Cayouette, IRCM, Montreal). GFP-Tubulin and GFP-UtrCH in the pCS2eGFP vector were kind gifts from W. Bement, University of Wisconsin, Madison.

### Transfections

HeLa cells were plated 1 day prior to transfection on 35 mm glass-bottom dishes (1.5 mm thickness; MatTek) in antibiotic-free MEM media. Transfections were performed using Effectene Transfection Reagent according to the manufacturer’s protocol (Qiagen). Complexes were washed out after 6 h to minimize cytotoxicity. To achieve maximal numbers of mitotic cells for imaging, transfections were performed at least 30 h before imaging. To detect microtubules and filamentous actin, cells were transfected with 200 ng GFP-Tubulin and GFP-UtrCH, respectively. For dominant negative experiments, cells were transfected with 300 ng of the relevant construct and transfected cells were identified by GFP expression from the bicistronic pCIG2.ires.eGFP vector. Transfected cells with an average GFP fluorescence intensity of <1400 arbitrary units were used for analysis.

### Immunofluorescence

For fixed confocal imaging, cells were grown on #1.5 coverslips. For super resolution microscopy, cells were grown on Zeiss high precision #1.5 coverslips (a kind gift from the CIAN microscopy facility) prepared for superresolution imaging by washing with spectroscopic grade ethanol and methanol. First, live cells were stained for mitochondria, then fixed with 3.2 % paraformaldehyde in phosphate buffered saline (PBS) for 10 min, permeabilized with PBS containing 0.1 % Triton X-100 and blocked with 2 % bovine serum albumin (BSA) in PBS for 30 min. Primary antibodies were incubated overnight at 4 °C and secondary antibodies were incubated for 45 min at room temperature in the dark. The following primary antibodies were used: rabbit anti-alpha-tubulin (Abcam, ab18251) and rabbit anti-RhoT1(A16) (Santa Cruz, sc-102083). Alexa Fluor 488- and 647-conjugated secondary antibodies were purchased from Invitrogen. DNA was stained with DAPI (Invitrogen). For fixed confocal microscopy, coverslips were mounted onto slides using PermaFluor (ThermoScientific). For superresolution SIM, coverslips were mounted onto slides with Prolong Gold (Invitrogen) and left to cure for 60 h.

### Microscopy and imaging

Fixed confocal imaging was performed using a Confocor LSM 510 META confocal system on a Zeiss Axiovert 200 M inverted microscope using a 1.4 NA 63× oil-immersion objective and Zen imaging software (Zeiss). MitoTracker Red fluorescence was excited with 514 nm laser, Alexa 488 fluorescence was excited with 488 nm laser and DAPI fluorescence was excited with 405 nm laser. Confocal stacks were acquired with a 0.3 μm step size.

Superresolution SIM was performed using an ELYRA PS.1 superresolution system combined with an LSM 710 laser scanning confocal microscope (Zeiss). Images were acquired using a 1.4 NA 63× oil-immersion objective and captured with a pco.edge sCMOS camera controlled by Zen imaging software (Zeiss). Alexa-488 fluorescence was excited with a 488 nm laser, MitoTracker Red fluorescence was excited with a 561 nm laser and Alexa 647 fluorescence was excited with a 642 nm laser. Emission was collected with the appropriate filter sets optimized for each laser line. Stacks were acquired with a 0.2 μm step size. SIM images were reconstructed from the raw data using Zen imaging software.

Live imaging was performed using a Quorum WaveFX-X1 spinning disk confocal system on a Leica DMI6000B inverted microscope (Quorum Technologies Inc.). HeLa cells were grown on 35 mm glass-bottom dishes (1.5 mm thickness; MatTek) and maintained on the microscope stage in complete phenol-free MEM media (Invitrogen) at 37 °C and 5 % CO_2_ using a Chalamide TC environmental control system (Live Cell Instruments). All Images were acquired using a 1.4 NA 63× oil-immersion objective and captured with a Hamamatsu ImagEM EM-CCD camera controlled with Metamorph software (Molecular Devices). MitoTracker Deep Red FM fluorescence was excited with a 643 nm laser and 25 ms exposure time and collected with an ET 700/75 emission filter set. GFP fluorescence was excited with a 491 nm laser line and 100 ms exposure time and collected with an ET 525/50 emission filter set. For time-lapse experiments, images were collected every 30 s. At each time point, 30 z-series optical slices were obtained with a step size of 1.0 μm using an ASI MS-2000 piezo stage.

### Image analysis

For each cell fluorescence intensities were systematically quantified both spatially (linescan from cell pole to cell equator; all four quadrants) and at five representative phases of division (metaphase to late cytokinesis) as described previously [15] with the following modifications: analysis was performed on average z-stack projections and a 40 pixel-wide linescan was used to measure average fluorescence intensity. Fluorescence intensity measurements from multiple cells were normalized and averaged using custom algorithms in MATLAB (mathworks) and transferred to GraphPad (Prism) for graph plotting. This analysis scheme allows for the direct and statistical comparison of average fluorescence intensity between division stages or culture conditions.

Linescan data were also used to calculate mitochondria and F-actin equator: pole fluorescence intensity ratios. Briefly, ratios were calculated using the first five (pole) and last five (equator) data points of each linescan at 30-s intervals from metaphase exit (t = 0 min) to late anaphase (t = 4.5 min) and then averaged across multiple cells.

The brightness, contrast and background were adjusted using Metamorph software (Molecular Devices). In order to aid the visualization of weakly fluorescent astral microtubules from live imaging data (Fig. 1a), the gamma was altered for presentation purposes, but was left unchanged for analysis.

### Statistics

Paired F-tests were used to compare mitochondrial distributions at five representative division stages in control cells versus KIF5BTail- or Miro-1ΔTM-expressing cells and DMSO- versus C3-treated cells. Mitochondria and F-actin distributions were also compared at five representative division stages using paired F-tests. All analyses were performed using GraphPad Software version 6.0 for Mac. Significance was set at p < 0.05.

## Additional files

10.1186/s13008-016-0015-4 Mitochondria and microtubules in dividing HeLa cells.
10.1186/s13008-016-0015-4 Metaphase mitochondrial distribution is not dependent on microtubules. HeLa cells in metaphase stained with MitoTracker Deep Red FM to visualize mitochondria and treated with either 0.02 % DMSO (control) or 10 μM Nocodazole to depolymerize microtubules. Shown are images of a single z slice from the centre of the confocal stack and a maximum z-stack projection.
10.1186/s13008-016-0015-4 Mitochondria in dividing HeLa cells expressing dominant negative KIF5B and Miro-1ΔTM constructs.
10.1186/s13008-016-0015-4 Quantification of mitochondrial distribution in HeLa cells transfected with control or wild-type, full-length Miro1. The mitochondrial fluorescence intensity from cell pole to equator was quantified in control pCIG2-expressing cells (5 cells, N=20) and full-length Miro1-expressing cells (10 cells, N=40) during metaphase, anaphase, and early-, mid- and late-cytokinesis. The normalized distance from cell pole to equator is displayed on the x-axis and the average fluorescence intensity normalized against the mean is displayed on the y-axis. Data are represented as the mean +/- SEM and lines fitted by non-linear regression. The difference in mitochondrial distribution was not statistically significant in Miro1-expressing cells compared with control cells (F-Test, p<0.05).
10.1186/s13008-016-0015-4 Mitochondria and F-actin in dividing HeLa cells.
10.1186/s13008-016-0015-4 Localization of mitochondria and cytoplasmic F-actin in control and aberrant cells. Spinning disk confocal images of HeLa cells transfected with GFP-UtrCH to visualize F-actin (green) and stained with MitoTracker Deep Red FM to visualize mitochondria (magenta). Representative images of cells in metaphase with either control, collapsed or aggregated mitochondrial phenotypes. Corresponding linescans are shown below the microscope images. The yellow and blue arrowheads indicate the colocalization of aberrant cytoplasmic F-actin and mitochondria in cells with the collapsed and aggregated phenotypes respectively. The yellow lines on the merge indicate the position of the linescans and black arrows on the linescans indicate mitochondria colocalized with cytoplasmic F-actin. Bar, 10 μm.
10.1186/s13008-016-0015-4 Mitochondria in dividing HeLa cells treated with C3 Transferase.

## Abbreviations

ROS: reactive oxygen species
Miro: mitochondrial Rho GTPase
SIM: structured illumination microscopy
F.I.: fluorescence intensity
