# Supplementary figures and images for: Mitochondria-cytoskeleton associations in mammalian cytokinesis
Source: Cell Div. 2016 Mar 18;11:3. doi: 10.1186/s13008-016-0015-4 (PMC4812650; doi:10.1186/s13008-016-0015-4)

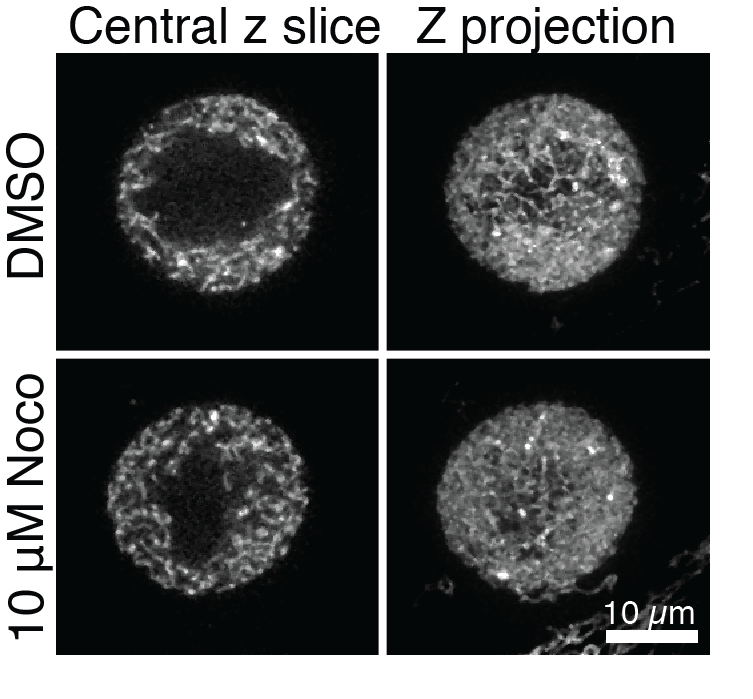

Supplement: Supplementary file 2 — 10.1186/s13008-016-0015-4 Metaphase mitochondrial distribution is not dependent on microtubules. HeLa cells in metaphase stained with MitoTracker Deep Red FM to visualize mitochondria and treated with either 0.02 % DMSO (control) or 10 μM Nocodazole to depolymerize microtubules. Shown are images of a single z slice from the centre of the confocal stack and a maximum z-stack projection. [file 13008_2016_15_MOESM2_ESM.tiff]

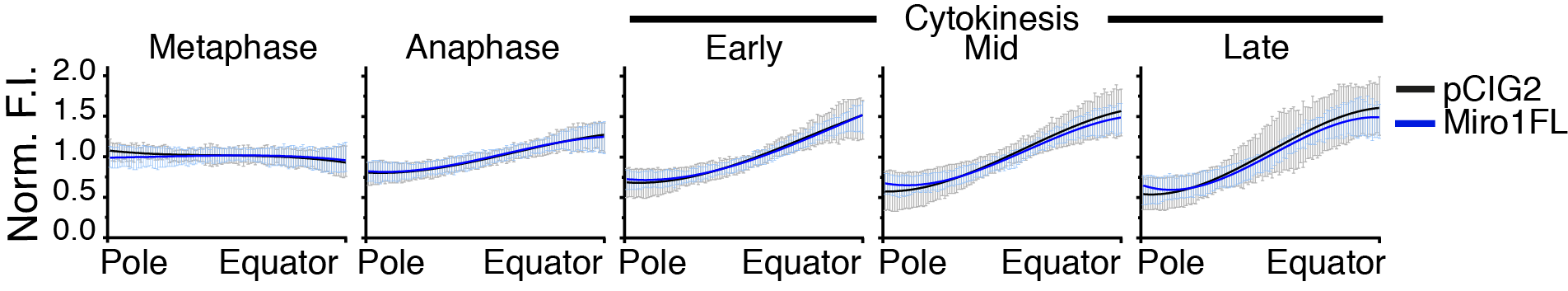

Supplement: Supplementary file 4 — 10.1186/s13008-016-0015-4 Quantification of mitochondrial distribution in HeLa cells transfected with control or wild-type, full-length Miro1. The mitochondrial fluorescence intensity from cell pole to equator was quantified in control pCIG2-expressing cells (5 cells, N=20) and full-length Miro1-expressing cells (10 cells, N=40) during metaphase, anaphase, and early-, mid- and late-cytokinesis. The normalized distance from cell pole to equator is displayed on the x-axis and the average fluorescence intensity normalized against the mean is displayed on the y-axis. Data are represented as the mean +/- SEM and lines fitted by non-linear regression. The difference in mitochondrial distribution was not statistically significant in Miro1-expressing cells compared with control cells (F-Test, p<0.05). [file 13008_2016_15_MOESM4_ESM.tiff]

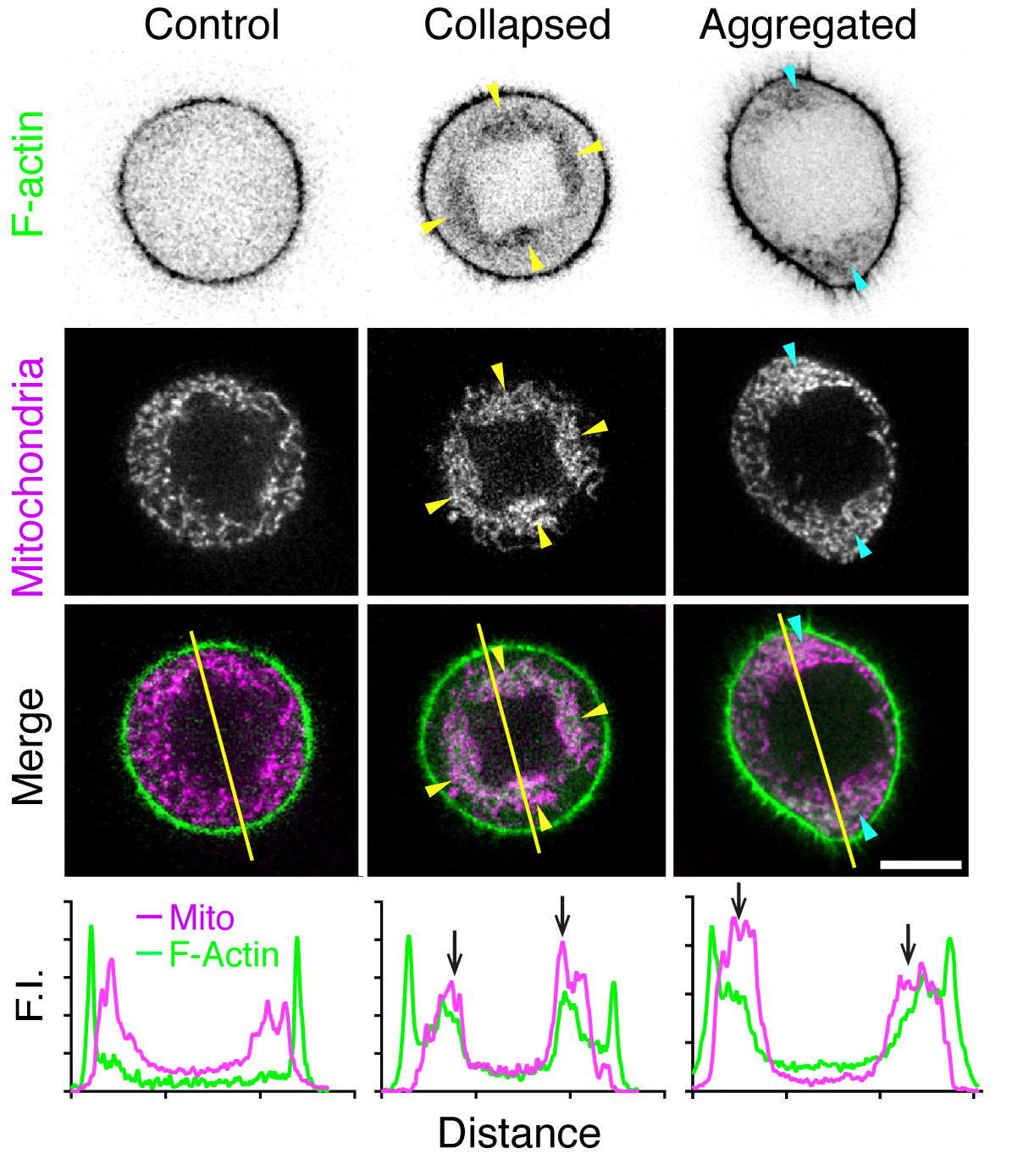

Supplement: Supplementary file 6 — 10.1186/s13008-016-0015-4 Localization of mitochondria and cytoplasmic F-actin in control and aberrant cells. Spinning disk confocal images of HeLa cells transfected with GFP-UtrCH to visualize F-actin (green) and stained with MitoTracker Deep Red FM to visualize mitochondria (magenta). Representative images of cells in metaphase with either control, collapsed or aggregated mitochondrial phenotypes. Corresponding linescans are shown below the microscope images. The yellow and blue arrowheads indicate the colocalization of aberrant cytoplasmic F-actin and mitochondria in cells with the collapsed and aggregated phenotypes respectively. The yellow lines on the merge indicate the position of the linescans and black arrows on the linescans indicate mitochondria colocalized with cytoplasmic F-actin. Bar, 10 μm. [file 13008_2016_15_MOESM6_ESM.tiff]
